# Dystocia, Delivery, and Artificial Intelligence in Labor Management: Perspectives and Future Directions

**DOI:** 10.3390/jcm13216410

**Published:** 2024-10-25

**Authors:** Antonio Malvasi, Lorenzo E. Malgieri, Michael Stark, Andrea Tinelli

**Affiliations:** 1 Unit of Obstetrics and Gynecology, Department of Interdisciplinary Medicine (DIM), University of Bari “Aldo Moro”, Policlinico of Bari, 70124 Bari, Italy; antoniomalvasi@gmail.com; 2 Chief Innovation Officer in CLE, 70126 Bari, Italy; lorenzo@malgieri.org; 3 The New European Surgical Academy (NESA), 10117 Berlin, Germany; mstark@nesacademy.org; 4 Department of Obstetrics and Gynecology and CERICSAL (CEntro di RIcerca Clinico SALentino), Veris delli Ponti Hospital Scorrano, 73020 Lecce, Italy

**Keywords:** dystocia, intrapartum ultrasound, artificial intelligence, prolonged labor, obstructed labor, cesarean section, operative delivery, complication, medical–legal claims, obstetrics malpractice

## Abstract

Labor management remains a critical issue in obstetrics, with dystocic labor presenting significant challenges in both management and outcomes. Recent advancements in intrapartum ultrasound have facilitated substantial progress in monitoring labor progression. This paper explores the integration of artificial intelligence (AI) into obstetric care, focusing on the Artificial Intelligence Dystocia Algorithm (AIDA) for assessing spatial dystocia during labor. The AIDA utilizes intrapartum ultrasonography to measure four geometric parameters: the angle of progression, the degree of asynclitism, the head–symphysis distance, and the midline angle. These measurements are analyzed using machine learning techniques to predict delivery outcomes and stratify risk. The AIDA classification system categorizes labor events into five classes, providing a nuanced assessment of labor progression. This approach offers several potential advantages, including objective assessment of fetal position, earlier detection of malpositions, and improved risk stratification, placing labor events within a broader context of labor dystocia and obstetric care and discussing their potential impact on clinical practice. This paper serves as a more comprehensive overview and discussion of the AIDA approach, its implications, perspectives, and future directions. However, challenges such as the technological requirements, training needs, and integration with clinical workflows are also addressed. This study emphasizes the necessity for additional validation across diverse populations and careful consideration of its ethical implications. The AIDA represents a significant advancement in applying AI to intrapartum care, potentially enhancing clinical decision-making and improving outcomes in cases of suspected dystocia. This paper explicates the key methodological approaches underpinning the AIDA, illustrating the integration of artificial intelligence and clinical expertise. The innovative framework presented offers a paradigm for similar endeavors in other medical specialties, potentially catalyzing advancements in AI-assisted healthcare beyond obstetrics.

## 1. Introduction

Labor is a complex physiological process with outcomes that can be challenging to predict. It does not always culminate in an unassisted delivery; often, it leads to either a vaginal or surgical birth intervention [1]. When labor becomes dystocic, complications arise. Dystocia, characterized by difficult or obstructed labor, encompasses a range of phenomena, from abnormal deceleration of cervical dilation or fetal descent during active labor to shoulder dystocia, an obstetric emergency in which the fetal shoulders become trapped after the delivery of the head [2]. Dystocia necessitates surgical intervention in 60–80% of cases and is a significant contributor to overall cesarean section rates. Medical management of dystocia is warranted only when the birth canal is fully dilated and after excluding abnormal fetal positioning, absolute or relative fetal macrosomia, and birth canal constrictions or deformities [3].

Labor dystocia, or failure to progress, is a primary factor necessitating a cesarean section. Defined as labor lasting significantly longer than expected (typically 20 h or more), prolonged labor can increase the risk of maternal and neonatal infections, fetal distress, neonatal asphyxia, uterine rupture, and postpartum hemorrhage. It may also indicate an increased risk of maternal pelvic floor and genital trauma during delivery [4,5].

Recent advancements in medical technology have introduced more objective and less invasive methods for labor monitoring. Intrapartum ultrasonography has shown significant utility in labor management, particularly in cases of dystocic labor. Researchers have proposed and evaluated various ultrasound parameters for assessing fetal head descent, progression, and internal rotation. These include the angle of progression, fetal head–symphysis distance, the midline angle, and the degree of asynclitism. The incorporation of asynclitism measurements into recent research has underscored its critical role in labor progression and potential complications.

Artificial intelligence (AI) has further enhanced the accuracy of diagnosing prolonged and dystocic labor, improving labor outcome assessments. An AI-driven approach known as the Artificial Intelligence Dystocia Algorithm (AIDA) combines multiple geometric parameters measured through intrapartum ultrasonography with machine learning techniques. This innovative method offers a more comprehensive assessment of labor progress and predictions of delivery outcomes, potentially revolutionizing intrapartum care and decision-making processes.

## 2. Intrapartum Ultrasound and Fetal Parameters

Monitoring the progress of labor is a critical aspect of intrapartum care, essential for ensuring optimal maternal and fetal outcomes. Traditionally, this process has relied heavily on clinical assessment, including vaginal examinations to determine cervical dilation, effacement, and fetal station.

Intrapartum ultrasonography has demonstrated significant utility in labor management over the past four decades, particularly in cases of dystocic labor [6,7]. This modality has shown marked advantages over traditional digital vaginal examinations [5]. Researchers have proposed and evaluated various ultrasound parameters for assessing fetal head descent, progression, and internal rotation, including measurement of the midline angle [8,9,10].

Furthermore, intrapartum ultrasound has substantially enhanced the diagnostic accuracy of certain fetal malpositions [11], notably asynclitism [12,13]. These conditions often prove challenging to evaluate through conventional vaginal examination due to the presence of caput succedaneum and molding, which can obscure key anatomical landmarks. A significant innovation in recent research has been the incorporation of asynclitism measurements [14,15]. These studies have underscored the critical role of this malposition which, when combined with other malpositions and malrotations, can significantly influence the progression of dystocic labor and potentially lead to maternal and fetal complications [16,17,18]. Malvasi et al. elucidated the physiological consequences of prolonged dystocic labor, particularly when precipitated by fetal head malpositions and malrotations. Their findings indicated that such conditions can result in lower uterine segment distension, ischemia, and apoptosis. Furthermore, they observed alterations in various neurofibrils, including enkephalinergic [19,20,21], adrenergic [22], and oxytocinergic [23] systems. These researchers also postulated potential effects on uterine pacemakers [24], although this aspect requires further investigation.

Recent advancements in artificial intelligence (AI) have enhanced the accuracy of diagnosing prolonged and dystocic labor, improved labor outcome assessments, and shown that combining multiple parameters provides better predictive accuracy for birth outcomes than analysis of individual parameters. The AI-driven approach known as the Artificial Intelligence Dystocia Algorithm (AIDA) [25,26] incorporates four key ultrasonographic parameters: the angle of progression (AoP) [27], fetal head–symphyseal distance (HSD) [28], the midline angle (MLA) [29], and the degree of asynclitism (AD) [30].

**Figure 1:**
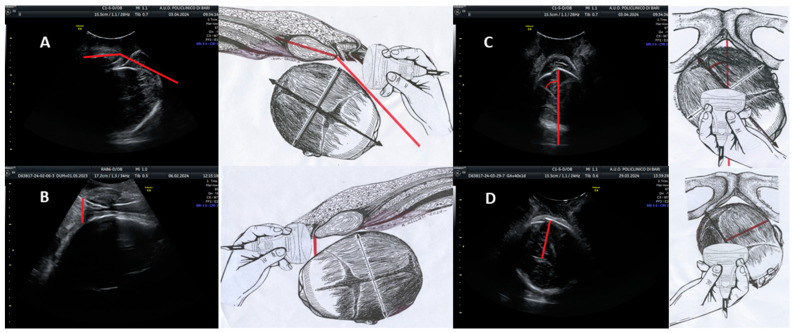
Ultrasound parameters for intrapartum assessment of fetal head progression and position. (**A**) Angle of progression (AoP): longitudinal scan showing the AoP (red line) with the fetal head in the occiput anterior position. (**B**) Fetal head–symphysis distance (HSD): sagittal ultrasound section aligned with the HSD along the infrapubic line (red line), with the fetal head in the occiput anterior position. (**C**) Midline angle (MLA): Transverse probe placement parallel to the bi-ischial line; ultrasound image shows the MLA (red angle) with the fetal head in the right occiput anterior position. Midline visible as an echogenic line between the cerebral hemispheres; the red line indicates the infrapubic line. (**D**) Asynclitism degree (AD): Transverse scan; the ultrasound image demonstrates AD measurement with the fetal head in the right occiput anterior position, showing anterior asynclitism. The red line perpendicular to the midline represents the degree of anterior asynclitism. Each parameter is illustrated with a schematic drawing (right) and a corresponding ultrasound image (left).

The angle of progression (AoP) is defined as the angle formed by a line passing through the midpoint of the pubic symphysis and a tangent to the fetal skull, as visualized on a longitudinal ultrasound scan. In Figure 1A, the schematic representation on the right and the corresponding ultrasonographic image on the left depict a longitudinal scan employed to evaluate the AoP. The red line demarcates this angle, with the fetal head positioned in the occiput anterior orientation.

The fetal head–symphysis distance (HSD) is evaluated using translabial ultrasonography with the patient in the lithotomy position, focusing on the median scan. This view enables the identification of two critical landmarks: the maternal pubic symphysis and the fetal head. The HSD is quantified as the minimum distance between the inferior margin of the maternal pubic symphysis and the fetal parietal bone. In Figure 1B, the right-hand drawing and the left-hand ultrasound image demonstrate a sagittal ultrasound section aligned with the HSD along the infrapubic line (highlighted in red). The fetal head is shown in the occiput anterior position.

The midline angle (MLA) is measured by assessing the angle formed between the midline of the fetal head (represented by the echogenic line separating the two cerebral hemispheres) and the anteroposterior diameter of the pubis. In Figure 1C, the right-side illustration indicates the correct probe placement, positioned transverse to the labia majora and parallel to the bi-ischial line, for MLA assessment. The corresponding ultrasound image on the left displays the MLA with the fetal head in the right occiput anterior position. The midline is visualized as an echogenic line separating the cerebral hemispheres, while the red line represents the infrapubic line. The angle formed between these lines, delineated in red, constitutes the MLA.

In the context of translabial ultrasonography, asynclitism is categorized as anterior when the fetal head midline deviates towards the sacrum and posterior when it shifts towards the pubis. The degree of asynclitism (AD) is quantified by measuring the distance, in millimeters, between the fetal head midline and the presenting parietal bone utilizing translabial ultrasound in the longitudinal plane. In Figure 1D, the right-side diagram illustrates the appropriate transverse positioning for AD evaluation. The ultrasound image on the left demonstrates measurement of the AD with the fetal head in the right occiput posterior position, exhibiting anterior asynclitism. The midline is again visible as the echogenic line between the cerebral hemispheres. The red line, perpendicular to the midline, represents the degree of anterior asynclitism.

This description provides a comprehensive overview of the ultrasound parameters used in labor assessment, correlating schematic representations with actual ultrasonographic images to elucidate the measurement techniques for each parameter.

## 3. Artificial Intelligence in Obstetrics: The AIDA

Artificial intelligence (AI) is increasingly transforming obstetric care, offering novel approaches to diagnosis, risk assessment, and treatment planning. Machine learning algorithms have been developed to analyze complex datasets, including ultrasound images, electronic health records, and maternal–fetal monitoring data. These AI systems show promise in predicting adverse pregnancy outcomes, optimizing labor management, and enhancing fetal surveillance. Deep learning techniques have been applied to interpreting fetal heart rate patterns and ultrasound images with remarkable accuracy. As AI technology continues to evolve, it has the potential to augment clinical decision-making, improve patient outcomes, and reduce healthcare disparities in obstetrics. However, ethical considerations and the need for robust validation studies remain crucial challenges in this rapidly advancing field.

Currently, the Artificial Intelligence Dystocia Algorithm (AIDA) represents a significant advancement in the application of artificial intelligence to intrapartum care. Developed through two key studies, AIDA 1 [25] and AIDA 2 [26], this innovative novel approach combines and integrates multiple geometric parameters measured through intrapartum ultrasonography with machine learning techniques and algorithms to assess labor progress and predict delivery outcomes. The selection of algorithms, methodologies for performance evaluation, and the metrics employed were meticulously delineated in the respective publications on AIDA 1 and AIDA 2.

These seminal papers provide comprehensive elucidations of the machine learning techniques utilized, the rationale underpinning their selection, and the rigorous performance metrics applied to assess their efficacy in predicting labor outcomes. The high values obtained demonstrate the robust discriminative capability of the algorithms chosen in distinguishing between different delivery outcomes, underscoring the potential clinical utility of the AIDA approach in obstetric decision-making.

In the following, we delineate several key methodological approaches that not only underpin the AIDA framework but may also serve as a source of inspiration for analogous applications in diverse contexts. These methodological elements exemplify the innovative integration of artificial intelligence and clinical expertise, potentially offering a paradigm for similar endeavors in other medical specialties or scientific domains. By explicating these methodological nuances, we aim to elucidate the transferable aspects of the AIDA approach, thus fostering interdisciplinary dialogue and the cross-pollination of ideas in the broader scientific community.

### 3.1. The AIDA Methodology

A two-step methodology was applied to the data sample. The initial step, the correlation analysis, employed Pearson’s correlation coefficient to ascertain that the four geometric parameters exhibited negligible or statistically insignificant correlations, ensuring each parameter contributed unique information regarding labor progression. The subsequent step, the machine learning algorithm selection, involved applying diverse supervised machine learning algorithms to the four geometric parameters in conjunction with physician-determined delivery outcomes. The predictive performance of each algorithm was quantified to identify the most efficacious models.

### 3.2. The AIDA Classification System

The classification system is predicated upon the identification of cut-off values for each geometric parameter associated with intrapartum cesarean delivery (ICD) and non-ICD outcomes. A decision tree algorithm was utilized to establish these cut-offs: for each parameter, values strongly associated with non-ICD outcomes were designated as “green”, those highly correlated with ICD were classified as “red”, and in cases where the data sample revealed intermediate ranges of uncertainty, a “yellow” designation was applied.

Having assigned a color to each individual value for the four geometric parameters for every parturition, the AIDA classification system employs a structured approach to categorizing each labor event into one of five distinct classes. This color-coded stratification of the geometric parameters facilitates a nuanced assessment of labor progression, enabling a more refined classification of each case. AIDA class 0 denotes all four parameters being within the green zone, indicating a high probability of non-ICD outcomes. AIDA class 1 indicates one parameter being in the red or yellow zone and three being in the green zone. AIDA class 2 signifies two parameters being in the red or yellow zone and two being in the green zone. AIDA class 3 represents three parameters being in the red or yellow zone and one being in the green zone. AIDA class 4 denotes all four parameters being within the red or yellow zone, suggesting a heightened likelihood of ICD.

### 3.3. The AIDA’s Prediction Performance

The integration of the delivery predictions obtained from the three best performing algorithms with the AIDA classification system yielded significantly improved results. A particularly salient finding was the algorithm’s high predictive accuracy for delivery outcomes, notably in AIDA classes 0 and 4. In AIDA class 0, characterized by all geometric parameters being within the green zone, the consistent prediction of non-ICD outcomes suggests its potential utility in identifying cases where intervention may be safely deferred. Conversely, the accurate prediction of ICD in AIDA class 4 cases could expedite decision-making for cesarean delivery, potentially optimizing maternal and fetal outcomes by mitigating the duration of prolonged, unproductive labor. The final methodological step entails employing the most effective machine learning algorithms for predicting delivery outcomes based on the four geometric parameters’ values with consideration of the relevant AIDA class. This approach enables clinicians to evaluate a prediction’s clinical reliability.

The remarkable predictive accuracy, particularly at the extremes of the AIDA classification spectrum, underscores the potential of this algorithmic approach to enhance clinical decision-making in labor management.

## 4. Potential Advantages of the AIDA in Dystocic Labor Management

Dystocic labor, also known as dystocia, broadly defined as difficult or obstructed labor, encompasses a range of conditions that impede the normal progression of labor and delivery as forms of difficult labor characterized by abnormally slow progress [31].

This condition can arise due to inefficient uterine contractions, abnormal fetal presentation, or other complications that impede the normal process of childbirth. Dystocia can lead to obstructed labor, where despite strong uterine contractions, the fetus cannot descend through the birth canal due to an insurmountable barrier, often occurring at the pelvic brim [32].

Several types of dystocia have been recognized in obstetrics, each with characteristics and management implications, and the classification may vary slightly depending on the medical literature or clinical approach: geometric dystocia [33,34,35,36,37,38], mechanical dystocia [6,39,40,41,42,43], dynamic dystocia [44,45], fetal dystocia [6,46], uterine dystocia [24,47], functional dystocia [48], soft tissue dystocia [40], compound presentations [49], maternal exhaustion dystocia [50,51,52], and labor dystocia [6,38].

Through its simultaneous measurement of four geometric parameters—the angle of progression, asynclitism degree, head–symphysis distance, and midline angle—the AIDA offers a comprehensive view of the spatial relationships between the fetus and the maternal pelvis, provides an objective assessment of the fetal position, and holds potential as a comprehensive tool for evaluating, directly or indirectly, various types of dystocia documented in the medical literature.

This approach provides a more detailed and accurate picture of the progress of labor or labor obstruction that might be missed by traditional assessment methods. By quantifying parameters like the degree of asynclitism, the AIDA may enable earlier detection of fetal malpositions that could lead to dystocia.

The AIDA classification system offers a nuanced approach to risk assessment, helping clinicians tailor their management strategies based on the likelihood of successful vaginal delivery or the need for cesarean section.

The high accuracy in predicting outcomes for AIDA classes 0 and 4 could be particularly useful in clear-cut cases, potentially reducing uncertainty in decision-making and improving the timing of interventions. For cases classified as AIDA class 0 (with all parameters in the green zone), the high negative predictive value for cesarean delivery could give clinicians more confidence in allowing labor to continue, potentially reducing unnecessary interventions. Conversely, in AIDA class 4 cases (with all parameters in the red or yellow zone), the high positive predictive value for cesarean delivery could facilitate earlier decision-making for surgical intervention, potentially reducing maternal and fetal morbidity associated with prolonged, obstructed labor.

By allowing repeated measurements over time, the AIDA could provide a more dynamic monitoring of labor progress in cases of suspected spatial dystocia. This could help differentiate between cases that are slowly progressing and those that are truly obstructed.

By providing detailed information about fetal position and its changes over time, the AIDA could enable more personalized management of labor, including tailored guidance on maternal positioning, the timing of interventions, and decisions about the mode of delivery.

The AIDA’s use of standardized ultrasound measurements could lead to more consistent assessment and management of spatial dystocia across different healthcare providers and settings.

While these potential advantages are promising, it is important to note that the AIDA is a decision support tool and should be used in conjunction with clinical judgment and other aspects of patient care.

Further research, including prospective validation studies in diverse populations, will be crucial to fully realize and confirm these potential benefits in clinical practice.

## 5. Challenges and Considerations for the AIDA

While the AIDA offers promising advantages in assessing spatial fetal dystocia, there are several challenges and considerations that need to be addressed for its effective implementation and use.

Technological requirements: Implementing the AIDA requires access to high-quality intrapartum ultrasound equipment and appropriate software. Not all healthcare facilities, especially in resource-limited settings, may have access to the necessary technology. This could limit the AIDA’s widespread adoption and potentially exacerbate healthcare disparities.

Training and expertise: Effective use of the AIDA requires proficiency in intrapartum ultrasonography and interpretation of the geometric parameters. Adequate training programs would need to be developed and implemented to ensure healthcare providers can accurately obtain and interpret the measurements. The AIDA system could serve as an excellent educational tool for obstetric residents and midwives. By providing objective measurements and clear risk stratification, it could help trainees develop skills in assessing fetal position and predicting labor outcomes.

As healthcare systems increasingly adopt electronic health records, the AIDA could be integrated to provide real-time risk assessment and decision support. This integration could improve documentation, facilitate consultations, and enhance overall care coordination. By providing objective measurements and standardized risk assessment, the AIDA has the potential to reduce disparities in care that may arise from variations in clinical experience or subjective assessments. The clear, quantitative measurements provided by the AIDA could facilitate better communication with patients about their labor progress and the potential need for interventions, potentially improving informed decision-making and patient satisfaction.

Integration with clinical workflows: Incorporating the AIDA into existing clinical workflows could be challenging. It may require changes to current practices and could potentially increase the time required for patient assessment, at least initially. Ensuring that the AIDA enhances rather than disrupts patient care is crucial.

Overreliance on technology: There is the risk that clinicians might become overly reliant on the AIDA’s predictions, potentially neglecting other important clinical signs or their own judgment. It is crucial to emphasize that the AIDA is a decision support tool, not a replacement for comprehensive clinical assessments.

Validation in diverse populations: The existing AIDA studies have focused on specific patient populations. Further validation is needed across diverse ethnic groups, body types, and clinical scenarios to ensure the algorithm’s accuracy and applicability in various settings.

Handling of edge cases: While the AIDA has shown high accuracy in predicting the outcomes for clear-cut cases (AIDA classes 0 and 4), its performance in intermediate cases (classes 1, 2, and 3) may be less definitive. Managing these edge cases and communicating uncertainty to both clinicians and patients could be challenging.

Real-time data processing: The AIDA requires real-time processing of ultrasound data. Ensuring that the system can provide timely results without delays, especially in rapidly evolving labor situations, is crucial.

Ethical considerations: The use of AI in clinical decision-making raises ethical questions. Issues of patient autonomy, informed consent, and the role of AI in healthcare decisions need to be carefully considered and addressed.

Data privacy and security: The AIDA requires the collection and processing of sensitive patient data. Ensuring the security and privacy of these data, in compliance with the relevant regulations (e.g., HIPAA in the US and the GDPR in Europe), is paramount.

Algorithmic bias: Like all AI systems, the AIDA could potentially perpetuate or amplify biases present in its training data. Ensuring that the algorithm performs equitably across different patient populations is crucial.

Continuous updating and maintenance: Medical knowledge and best practices evolve over time. The AIDA will need to be regularly updated to reflect the latest evidence and clinical guidelines, which will require ongoing resources and expertise.

Cost-effectiveness: While the AIDA has the potential to improve care, its cost-effectiveness needs to be demonstrated. The initial investment in technology and training, as well as ongoing costs, needs to be balanced against potential improvements in outcomes.

Resistance to change: Healthcare providers may resist adopting new technologies, especially if they perceive them as challenging their clinical expertise or autonomy. Overcoming this resistance through education and demonstration of the AIDA’s benefits will be important.

Interpretability of AI decisions: The “black box” nature of some machine learning algorithms can make it difficult to understand how the AIDA arrives at its predictions. Developing explainable AI techniques to make the AIDA’s decision-making process more transparent is crucial to building trust and facilitating clinical adoption. The classification system comprising the five AIDA classes has the potential to substantially aid in addressing this challenge.

Handling of conflicting information: In cases where the AIDA’s predictions conflict with clinical impressions or other assessment tools, clear guidelines would be needed on how to reconcile these differences and make final management decisions.

Impact on the patient–provider relationship: The introduction of AI into the intimate setting of labor and delivery could potentially affect the patient–provider relationship. Ensuring that the AIDA enhances rather than detracts from personalized, compassionate care is important.

Cross-platform compatibility: Ensuring the AIDA can function across different ultrasound machines and healthcare IT systems is crucial for its widespread adoption.

Regulatory approval: As a medical device incorporating AI, the AIDA would need to navigate complex regulatory landscapes, which can vary by country and may not yet be fully developed for AI in healthcare.

Management of expectations: While the AIDA shows promise, it is important to manage expectations about its capabilities. Overpromising could lead to disappointment and erosion of trust if the system does not perform as expected in all scenarios.

## 6. AI, the AIDA, and Future Perspectives

The potential of the AIDA extends beyond just diagnosis. Future iterations could incorporate additional parameters such as maternal pelvimetry, contraction patterns, and maternal pushing efforts to provide a more comprehensive labor assessment tool. Integration with electronic health records could allow for real-time risk stratification and clinical decision support. As we move towards an era of AI-assisted healthcare, it is crucial that we continue to critically evaluate these promising tools, ensuring that they support rather than supplant clinical expertise and that they ultimately contribute to improved patient care and outcomes. The AIDA method offers a glimpse into the future of obstetric care, where advanced technology and clinical expertise work hand in hand to provide the best possible outcomes for mothers and babies.

Future research directions for the AIDA could include the following: (1) Large-scale, prospective validation studies across diverse healthcare settings and patient populations; (2) investigation of the AIDA method’s performance in different clinical scenarios, including for multiparous women and labors of a shorter duration; (3) the integration of additional clinical parameters to potentially enhance the predictive accuracy of the algorithm; (4) evaluation of the impact of implementation of the AIDA on clinical outcomes, including cesarean delivery rates, maternal and neonatal morbidity, and patient satisfaction; (5) cost-effectiveness analyses to assess the economic implications of implementing the AIDA in routine clinical practice; and (6) exploration of the potential role of the AIDA in reducing healthcare disparities by providing more objective assessment tools.

## 7. Conclusions

In conclusion, the AIDA method represents a significant advancement in the application of AI [53] to intrapartum care. By providing a more objective and comprehensive assessment of labor progress and its contribution to a potential diagnosis of suspected dystocic labor, it has the potential to enhance clinical decision-making and improve outcomes in cases of suspected dystocia. However, its integration into clinical practice should be approached cautiously, with ongoing evaluation and refinement based on larger-scale studies and real-world experiences of its implementation.
